# Leisure and cultural identity: an empirical study based on root-seeking summer camp for ethnic Chinese new generation

**DOI:** 10.3389/fpsyg.2024.1330613

**Published:** 2024-10-30

**Authors:** Zhenhan Wang, Sheling Ye, Li Bei

**Affiliations:** ^1^School of Philosophy, Zhejiang University, Hangzhou, China; ^2^Hangzhou International Urbanology Research Center and Zhejiang Urban Governance Studies Center, Hangzhou, China; ^3^School of Humanities, Wenzhou University, Wenzhou, China; ^4^School of Foreign Languages and Tourism, Wuxi Institute of Technology, Wuxi, China

**Keywords:** leisure, cultural identity, ethnic Chinese new generation, root-seeking, summer camp

## Abstract

This study examines the role of root-seeking summer camps in enhancing Chinese cultural identity among the new ethnic Chinese generation (ECNG) amidst the challenges of globalization. Semi-structured interviews with thirty campers revealed that ECNG often lack clear ethnic consciousness, experience conflict in community relationships, and have limited opportunities for traditional cultural experiences. The study found that summer camps exert both intrinsic and extrinsic influences on campers. Intrinsically, they facilitate the internalization of leisure motivation, foster a deepened leisure experience, and promote strengthened leisure interactions. Extrinsically, they provide opportunities for leisure education and cultural immersion. Through this synergy, summer camps enhance the cultural identification of ECNG. However, the study also noted that course enjoyment and teaching staff behavior could influence perceptions of the camp. This study contributes to understanding the relationship between leisure and identity, explaining how summer camp activities enhance ECNG’s cultural identity, and offering insights for governments and agencies.

## 1 Introduction

The global migration wave significantly impacts ethnic and cultural identity, especially for the new generation of global immigrants who are “wanderers” between the cultural differences of their original countries and the countries they now reside in. Many scholars have suggested that identity is variable (Xie, 2010), second-generation identity is complex and diverse (Li and Yao, 2012), and the new generation of Chinese differs greatly from the older generation of overseas Chinese in terms of political identity, identity awareness, and cultural identity (Yu, 2005). Therefore, Enhancing migrants’ cultural identity is an urgent and challenging task. Instead of focusing on the typical narrative of assimilation into host countries, this paper scrutinizes their relationship with cultural identity in their countries of origin to enhance their sense of cultural self-awareness. Using the experiences of the ECNG who participated in root-seeking summer camps as an example, it studies the possible impact of these camps on cultural identity, as well as the mechanisms of cultural expression and identification hidden behind leisure motivations and behaviors.

While previous studies have mainly focused on how immigrants integrate into host societies, the novelty of this research lies in its focus on how the new generation of ethnic Chinese (ECNG) reassesses and establishes their cultural identity through leisure activities. By examining the role of root-seeking summer camps, this study contributes a new perspective to the growing body of literature, highlighting the importance of leisure activities in shaping and strengthening cultural identity. The research reveals how carefully designed cultural experiences can foster a sense of identity and belonging among the ECNG. The findings provide fresh insights into the processes of cultural identity formation, showing that these cultural activities (such as summer camps) are not only significant on a recreational level but also support the construction of cultural identity on psychological and social levels.

Local overseas Chinese associations and primary communities hold various Chinese cultural leisure and experience activities for the ECNG to enhance their Chinese cultural identity, making them true promoters of cultural exchange and mutual learning. For example, the “China root-seeking Summer Camp” originated from “Study Tour” in 1980, and was officially named the “Overseas Chinese (and Hong Kong, Macao, Taiwan) Youth China root-seeking Summer Camp” in 1999. In 2010, Xi Jinping proposed the goal of achieving and continuing the roots of the Chinese nation, the soul of Chinese culture, and the dream of great rejuvenation at the opening ceremony of the root-seeking Summer Camp. Currently, more than 300,000 young people from over 100 countries and regions, including Hong Kong, Macao, and Taiwan, have participated in this activity.

In this article, the term “ECNG” is used as a unit to explore the cultural identity of Chinese descendants similar to the 1.5 generation, second generation, and third generation, as compared to the “first generation” immigrants (Bartley and Spoonley, 2008; Ren, 2021). Generally, the “Ethnic Chinese New Generation” refers to people who are mostly born outside of China. During their upbringing, they have been influenced by Chinese culture from their parents or grandparents, as well as by the local culture. They may not be as traditional as the older generation, and their language abilities and cultural customs may be different, but they still maintain a certain familiarity with Chinese culture (Schiller et al., 1995; Mannheim, 1952; Wang and Huang, 2023).

The purpose of this study is to delve into the intrinsic mechanisms by which ancestral heritage summer camps enhance the Chinese cultural identity among the ENCG. It aims to provide decision-making recommendations for the government, immigrant-related organizations, and communities, to harness the potential of root-seeking summer camp. By focusing on the transformative potential of cultural leisure activities, this study expands our understanding of how identity is negotiated and reinforced in a globalized context.

## 2 Literature review

### 2.1 Leisure and summer camps

Leisure is an ancient and complex concept. Since the publication of “The Theory of the Leisure Class,” scholars have had various definitions of leisure from different perspectives, such as free time, mindset, state of being, and activities (Godbey, 1994). Chinese scholars such as Pang Xuequan, Pan Liyong, and Ma Huidi believe that leisure is closely related to a way of life, a free experience of life, and a beautiful spiritual home for humanity.

Summer camps are generally seen as an extension of educational activities on campus with educational value. “Ci Hai” and “Xinhua Dictionary” generally emphasize that summer camps have the characteristics of “summer vacation” and define “camp” as a place for organizing meaningful series of activities. The American Camp Association, established in 1910, emphasizes that summer camps refer to organizing outdoor creative, entertaining, and educational activities during holidays.

Summer camps are a popular leisure activity for young people in summer, with positive implications for social integration, intellectual growth, and physical exercise. In the United States, children from all over the country go to various camps during the summer. Thurber et al. (2007) studied 3,395 children from 80 different day camps or residential summer camps and found universal growth in positive identity, social skills, physical and thinking skills, positive values, and spirituality. Fields (2008) believes that participating in summer camps can enhance peer relationships, personal autonomy, positive relationships with staff, and deepen scientific knowledge. Baker et al.(2019) believe that summer camps are traditionally linked to recreational activities and are a positive source of personal, physical, and moral development for children and adolescents. Barcelona et al. found that campers who participated in a summer day camp program reported that their interest in academic subjects increased throughout the camp. Furthermore, campers with higher levels of connectedness to camp reported significantly stronger academic and youth development outcomes than those with lower levels of connectedness (Barcelona and Hartman, 2021).

The root-seeking summer camp has developed into a program covering rich cultural experiences in addition to Chinese language education, aiming at “inspiring Ethnic Chinese youth’s interest in learning Chinese language and culture, enhancing their awareness and emotional connection to the ancestral (homeland) country (Li, 2020).” Xia (2020) believes that Chinese language summer camps can influence and enhance the cultural awareness, cognition, and identity of the ECNG by exposing participants to cultural knowledge and Chinese experiences, thereby enhancing their centripetal and cohesive forces with China and better presenting Chinese stories to worldwide audiences. However, there are also some critical voices about summer camps, for “summer camps have become training classes, and campers cannot truly relax and rest (Xie, 2006)” and “occasionally negative results are observed, including anxiety and boredom (Tong et al., 2020).” Overall, as a leisure activity, summer camps have a relatively positive impact and significance.

### 2.2 Leisure and cultural identity

The term “identity” stems from the Latin word idem, which means “the same” or “identical.” In English, it refers to the concept of “identity” as well as “equivalence” or “sameness.” Scholars define identity as “a set of meanings applied to the self in social roles or situations (Burke and Tully, 1977).” Some researchers argue that identity varies at micro and macro levels. The former is a persistent source of human behavior and motivation that reinforces individuals’ self-perception, while the latter connects individuals with the most general social meanings (Zhang, 2001). Identity includes various types, such as “racial identity, ethnic identity, social (group) identity, self-identity, and cultural identity, cultural identity is the core type of identity”(Cui, 2004). Stryker and Burke (2000) believe that people possess as many selves as the number of social groups with whom they interact. Hirsh and Kang (2016) suggest that given the increasing complexity of the social world, however, individuals are more and more likely to identify strongly with multiple social groups simultaneously. When these groups provide divergent behavioral norms, individuals can experience social identity conflict.

Cultural identity often involves nationality, ethnicity, religion, and social class, as these identity objects more or less contain cultural factors. Even the self-concept, which is inseparable from identity, is fundamentally a product of culture (Cui, 2004). Individuals confirm their unity of self-based on culture as the basic element. Therefore, this article attempts to present a comprehensive overview of the cultural identity of the ECNG based on cultural elements closely related to Chinese characteristics, such as their sense of community belonging, social interaction, traditional cultural experience, and so on.

With the global wave of immigration in the latter half of the 19th century, research on culture and self-identity among immigrants has been ongoing in academia. Many scholars focus on how to help immigrants identify with the ethnicity and culture of their host country thus better integrating it into the local way of life, to build a sense of cultural identity between immigrants and local culture. Through a study of a 15-year-old Chinese boy’s identity crisis, Luk (1993) detailed the issue of cultural identity among second-generation immigrants and pointed out the significant role of cultural factors in the crises and barriers to identity formation in adolescence. By studying the life experiences of Greek-Canadian and Jewish-Canadian immigrants who grew up in Halifax, Michele and Evangelia (2008) identify that specific settlement locations have important impacts on ethnic and cultural identity. McLean et al. (2018) believe that cultural background is crucial in personality and identity development, with identity development being restricted by the culture in which it is developed. Sharp et al. (2021) suggest that promoting parenting behaviors that encourage identity exploration through leisure experiences could be a promising avenue for fostering identity development. Wearing et al. (2021) suggest that gaming can create experiences that lead to the formation of self-identity through social interactions and relations that have the potential to build social and cultural capacities for adolescents while also enabling resistance to social norms. Case studies of overseas Chinese suggest that the local cultural identity of the ECNG is strengthening while the ancestral cultural identity is weakening compared with the older generation of Chinese immigrants. Dong also suggests that new strategies, such as enhancing China’s soft and hard power, strengthening rhetorical strategies, localizing Chinese language education, and shaping a more attractive ancestral culture, are needed to construct a new form of cultural identity for the ECNG (Dong, 2016).

With the deepening of China’s reform and opening up and the trend of the global market economy in the 1990s, Chinese scholars have realized that Chinese culture is faced with an “identity crisis” (Li, 1998; Liu, 1999; Ma, 2000) under the context of globalization and postmodernity, especially regarding the identity of overseas Chinese. Chinese scholars mainly focus on the characteristics and influencing factors of the cultural identity of overseas Chinese, as well as how to enhance cultural identity. Overall, these studies mainly involve the following aspects, characteristics of the cultural identity of overseas Chinese, which are constrained by multiculturalism (Kou, 2007), have multiplicity (Han, 2009), different attitudes (Li, 2017), etc.; influencing factors such as mastery and use of mandarin (Zhang and Lu, 2011), traditional Chinese festivals (He, 2008), cultural policy, institutional discrimination, media, education (Liu, 2002), generational differences (Dong, 2016), and national differences (Shen, 2017); path to enhance cultural identity. Most scholars believe that cultural identity can be enhanced through traditional dance (Yang, 2007), music (Hu, 2010), leisure sports (Zhan, 2011), and so on.

Many scholars examine the relationship between leisure and personal identity, social identity, and cultural identity from variables such as economic and social status, ethnic differences, and social roles. Most scholars believe that leisure activities enhance the shaping and development of identity. Zhong and Lin (2014) point out that leisure activities are related to subjective self-identity feelings. Walker et al. (2007) believe that active leisure participation enhances an individual’s sense of belonging to the community. Kleiber et al. (2014) believe that engaging and exploring leisure experiences may influence identity because individuals need self-expression and interaction with others, and leisure is considered to provide opportunities and contexts for individual interest expression. Haggard and Williams (1992) believe that through participation in certain leisure activities, people express and confirm an identity consistent with a set of identity images related to the selected leisure activity. However, some scholars believe that leisure hinders the development of identity. Shaw et al. (1995) believe that the mass media of globalization (such as watching TV for leisure) may hinder the development of identity. Piko and Vazsonyi (2004) use the term “leisure activity equalization” to demonstrate that Hungarian youth in the post-socialist era have become products of globalization, with high similarities to their Western peers. Yu and Berryman (1996) found that the leisure activities of the new generation of minority groups are likely to be assimilated into local culture, rather than enhancing their original cultural identity.

In summary, the issue of cultural identity among immigrants has been widely discussed in previous research, especially regarding the ethnic identity and cultural adaptation of second-generation immigrants. The importance of leisure activities in cultural identity has gradually been recognized, and leisure has been viewed as one of the most important ways to enhance cultural identity. Therefore, the purpose of this study is to explore the internal mechanism of how the root-seeking Summer Camp enhances the cultural identity of ECNG in light of leisure identity theory, thus providing references for various levels of authorities and communities, and enhancing cultural identity among ECNG through root-seeking summer camp.

## 3 Research methodology

### 3.1 Method

As this research regards root-seeking summer camp as a leisure activity for the ECNG, the activity is understood as a process of constructing cultural identity, while exploring the relationship between this leisure activity and the Chinese cultural identity. Thus, a semi-structured interview approach with a weak structure was adopted. This research is designed upon a constructivist approach, and each interview data is collected through direct dialog. The conversation process is not simply a “question and answer” format, but rather an overall observation and recording of interviewee’s subjective perceptions of their life background, cultural narrative, and other aspects during the answering process. The information obtained based on this is actually “a special conversation that creates meaning between the interviewer and interviewee, and generates knowledge (Hesse-Biber and Leavy, 2006).” Referring to Cheek’s (Cheek et al., 2002) aspects of identity questions, and considering that the object of this paper is the summer camp activities of teenagers, this article adopts the relevant questions such as “activity preference and experience” from the interview questionnaire by Fields (2008) and Thurber et al.’s (2007). The questions of leisure motivation and cultural experience, such as leisure motivation, leisure expectations, communication and meaning, etc., are designed to meet the leisure and cultural identity research needs of this paper.

The interview outline consists of two parts. The first part is the socio-demographic information of the interviewees, including age, country, and city of birth or residence, the generation of immigrants, and the number of times participating in summer camp activities. The second part includes 9 open-ended structured questions: (1) Can you tell us why you participated in the summer camp? (2) What are your expectations for this summer camp? Have your expectations been met? (3) Could you describe your favorite course and the most difficult project during the summer camp? (4) Could you tell us the happiest and most difficult things for you during this summer camp? (5) What do you think of the teachers (staff) here? (6) Describe what you did during the summer camp and which activities were the most important to you. (7) How was your overall experience here? (8) Are you willing to participate again in the future? Why? (9) Was this summer camp meaningful to you? If so, please describe the most impressive gains from the summer camp as detailed as possible.

### 3.2 Sampling

The research samples of this study are from the root-seeking summer camp organized by Wenzhou University. Considering that Chinese immigrants to overseas countries mainly come from specific provinces such as Zhejiang Wenzhou city, local overseas Chinese associations, and primary communities often bear responsibility for organizing summer camp activities. Meanwhile, the root-seeking summer camp projects are generally managed by local educational institutions. Known as a hub for overseas Chinese immigrants, Wenzhou in Zhejiang Province has a rich immigrant history dating back to the 1980s. Many immigrants send their children back to China for summer vacations. In response to this trend, Wenzhou overseas Chinese associations and local communities independently organize enriching cultural activities aiming to enhance the summer experiences of these young Chinese descendants. Over the past two decades, an annual root-seeking summer camp organized by Wenzhou University has been welcomed by young overseas Chinese worldwide. Each summer, the camp hosts a variety of young overseas Chinese individuals, offering them a plethora of cultural activities ranging from traditional Chinese paper cutting, calligraphy, Han-era ceremonies, the intangible cultural heritage of Wenzhou, pastry making, and martial arts. This gamut of cultural activities is gradually exerting a growing impact on the communities of the ECNG.

In 2019, a total of 232 Chinese youth campers from 14 countries participated in the program, The summer camp set up five different themed projects: Chinese Leaders Camp, Chinese Kongfu Camp, Chinese Music Camp, Chinese Talent Camp, and Chinese Design Camp, which campers could choose from according to their interests when registering. Each project has its unique cultural curriculum, which includes classroom lectures, museum visits, and cultural activities like traditional handicraft experiences. The camp activities are scheduled in three-time slots daily: (9:00–11:30), (14:30–17:20), and (19:00–20:30), with the remaining time for meals and personal time. Campers need to complete a 12-day curriculum and stay in a hotel arranged by the program, one of which is on campus and another 2 km away from campus. The following lists the main featured courses of the Talent Camp and Kongfu Camp (each theme has designed special courses and public courses) Table 1. These courses are guided by different teachers for the campers to complete.

**Table 1:** Partial root-seeking summer camp courses.

Projects	Special courses		Public courses
Chinese talent project	Paper cutting	Appreciation and learning of Wenzhou paper cutting art, involving hands-on student practice	Singing Contest (2 days) Visit to beautiful rural areas Understanding of Wenzhou culture from geographical and historical perspectives Sino-Western music appreciation concert Viewing of the movie “A Bite of China” Visit the “World Wenzhou People Museum” Morning reading (every day) Sports activities (every day)
	Chinese Characters	Understanding how Chinese characters are constructed through hands-on exercises	
	I am a speaker	Exploration of the significance of public speaking with a focus on hands-on 1-min self-introduction speech	
	Calligraphy	Overview of Tang Dynasty calligrapher Yan Zhenqing, with interactive student calligraphy practice	
	Pottery	Introduction to fundamental tools and techniques of pottery making with hands-on experiences	
	Chinese Painting	Basic knowledge acquisition of Chinese painting, including creating a small artwork through active student participation	
	Peking Opera Facial Makeup	Appreciation of Chinese Peking Opera excerpts, followed by hands-on creation of facial makeup expressing diverse characters	
	Chinese Folk Songs	Understanding of Chinese folk songs with hands-on learning to perform the Chinese folk song “Jasmine”	
	Hanfu (Han clothing)	Appreciation and exploration of Han ethnic costumes, involving hands-on activities such as trying on the costumes	
Chinese Kongfu Camp	Basic Kongfu Skills	Hands-on practice of martial arts long fist techniques	
	Kickboxing	Hands-on practice of martial arts long fist techniques	
	Dragon Dance	Fundamental techniques and footwork of dragon dance, practice with an emphasis on active student involvement.	
	Chinese Traditional Culture	Calligraphy, Chinese painting, Paper cutting, Guzheng (Chinese zither), Hanfu Visit Kongfu Museum	

For this study, 30 Chinese youths from the five camps were randomly selected for in-depth interviews. We randomly select participants within the camp. Normally, we will wait at any other appropriate locations outside the classroom, in the cafeteria, or after their activities or courses end. If we are not in the classroom, we will wait in the hotel lobby for their tour activities to end. If the participants have ample time for meals, rest, or free time, we will randomly select a participant. After informing them of the purpose of our face-to-face interview, confidentiality, and obtaining their consent, we will start in-depth one-on-one face-to-face interviews. These interviews usually last 30 to 60 min. Such an interview model ensures that we can obtain unique and original data, which helps us to deeply understand the thinking patterns and responses of the experimental subjects. The socio-demographic information of the interviewees is shown in Table 2. The real names were omitted to protect the participants’ privacy.

**Table 2:** Socio-demographic information of participants.

Participant	Age	Generation immigrants	Nationality	Participant	Age	Generation immigrants	Nationality
P1	15	2	Austria	P16	17	2	Portugal
P2	17	2	Austria	P17	16	3	Italy
P3	14	2	Austria	P18	17	2	Italy
P4	14	Unknown	Germany	P19	15	3	Greece
P5	14	Unknown	Portugal	P20	16	2	Greece
P6	12	2	Austria	P21	17	3	Italy
P7	13	2	Austria	P22	16	2	Russia
P8	12	Unknown	Portugal	P23	14	2	Greece
P9	16	2	Belgium	P24	14	2	Italy
P10	14	Unknown	America	P25	15	2	Italy
P11	15	2	Greece	P26	14	2	Italy
P12	17	2	Spain	P27	15	2	Italy
p13	17	2	Spain	P28	16	2	Germany
P14	13	Unknown	America	P29	15	2	Russia
P15	16	3	France	P30	17	2	Portugal

### 3.3 Data analysis

All interviews were recorded with audio devices. 28 interviews were conducted in Chinese, one interviewee primarily spoke English with Chinese as a secondary language, and one interviewee spoke German with the help of a Chinese translator to avoid typos and omissions. Data analysis was conducted by all the authors together, including transcription, proofreading, and text analysis. Firstly, a careful study of the data to see what was happening to the respondents was done. To take an analytic stance, the initial coding was done line-by-line without preconceptions, trying to keep necessary information such as tone, intonation, mood, laughter, etc., as much as possible, to ensure the accuracy of transcription. The next procedure was developing conceptual codes to categorize the initial codes together with related information in each interview first and then summarize the similar experiences of the respondents and use Excel to extract the initial themes. Finally, a leading code was developed to embrace all the related sub-categories. Comparison work between data with data, data with category, and category with category to observe similarities and differences between the respondents was done throughout the coding process and a theoretical model was built at last.

## 4 Results

**Figure 1:**
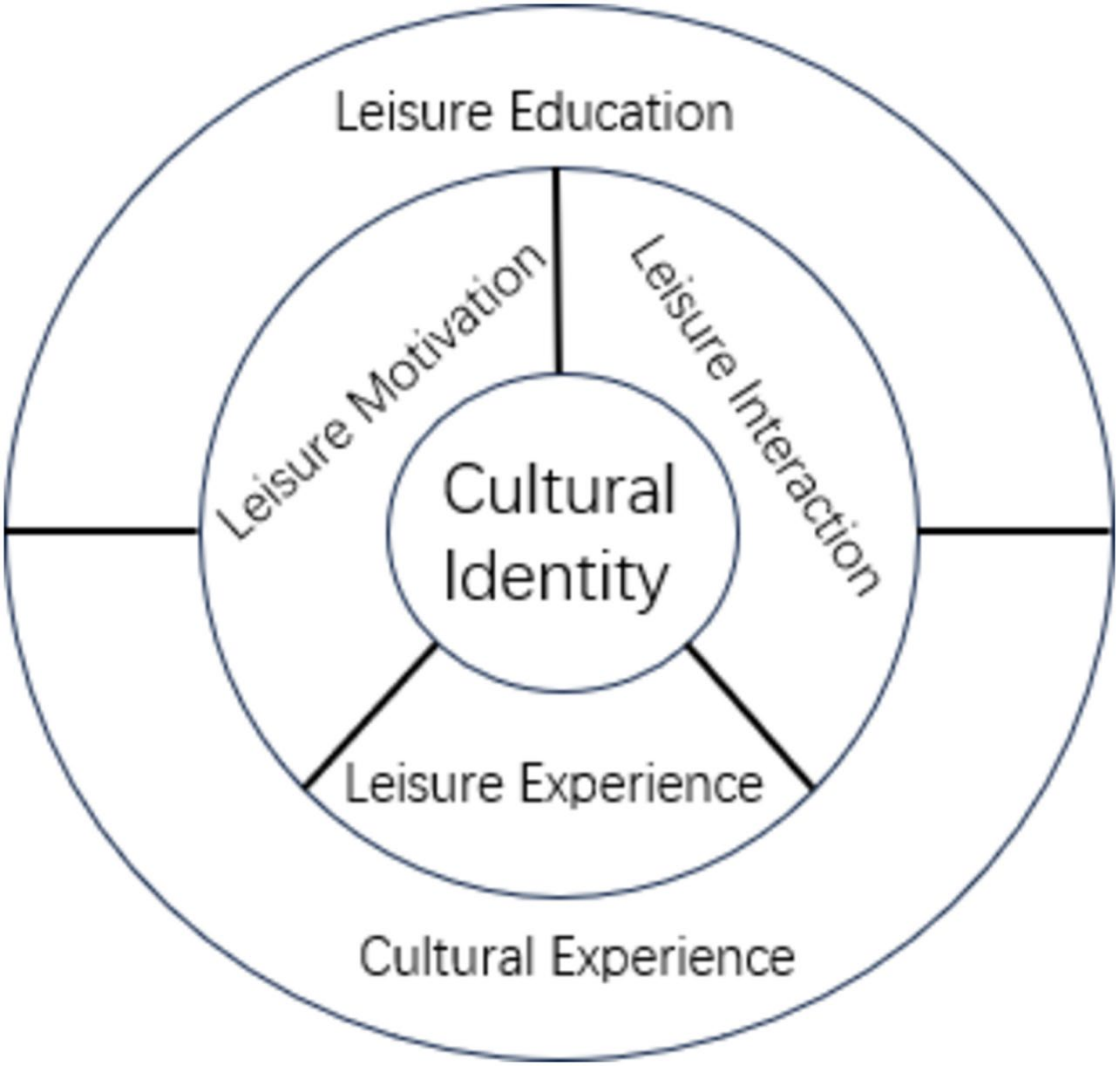
The mechanism of summer camp enhances cultural identity.

The data presents that the ECNG exhibits a rather insufficient ethnic awareness, social conflicts exist in community interactions, and a lack of opportunities to experience traditional culture. The research underscores that the summer camp, manifested as a leisure activity, is conducive to enhancing the clarity of ethnic consciousness through the fortification of Chinese cultural leisure motivation. The incorporation of participatory leisure experiences aids in creating opportunities for immersion in traditional Chinese culture, counteracting its experienced scarcity. Bringing together campers sharing similar cultural backgrounds to engage in leisure activities has the potential to alleviate social conflicts within the community. The three constituents - “leisure motivation, leisure experience, and leisure interaction,” ostensibly construct the implicit sensations of leisure within the campers, influencing their identification with Chinese culture through innate and nuanced mechanisms (Figure 1).

Subsequently, the overall summer camp activity is carried out within a prepared environment of leisure education and an abundant dose of traditional cultural experience. The ensuing cultural identity is not left to chance but is fostered through strategically chosen conventional cultural formats from the campers’ hometowns, in educationally prepared settings, engaging the campers in an intensive traditional cultural experience process. Undeniably, the elements of leisure education and cultural experiences require a considerable amount of conscious preliminary planning by the organizers, rendering a noticeable guiding effect on the campers’ internal sensations and cultural identification.

Overall, through the campers’ participation in heritage tour summer camp leisure activities, the integration of the tension and conflict between Chinese culture and their host country’s culture is facilitated. In a state of free choice, free expression, free experience, and a relatively pressure-free environment, the tensions and conflicts associated with their ethnic consciousness, community relations, and opportunities for cultural experience noticeably ease off, enhancing their identification with Chinese culture. However, it’s also noted that the degree of course fun and the behavior of the teaching staff may generate pessimistic perceptions of the summer camp.

### 4.1 Characteristics of Chinese culture in the ECNG

#### 4.1.1 Insufficient ethnic awareness

Developmental psychology suggests that cultural identity begins to form in childhood. Specifically, individuals usually explore their ethnicity in early adolescence and form a relatively stable ethnic identity around the age of 17. The vast majority of second-generation Ethnic Chinese are born or educated in other countries. They can distinguish their nationality and ethnicity more clearly and can explain what “Chinese people” are and how they are related to “China.” Most interviewees believe that Chinese people should have a blood relationship and be able to speak Chinese. P3 mentioned: “I am also Chinese. My appearance and the fact that I can speak Chinese make me Chinese.” P5 pointed out: “I am Chinese because our fathers or grandfathers are also related to Chinese people.”

However, some interviewees identify themselves as Chinese but cannot identify other campers as Chinese. P16 said: “I am Chinese, I am very Chinese…. Many foreign children (referring to other campers) grew up outside and were exposed to little Chinese culture. They are a bit different, but they know they are Chinese. They may come here and have a better understanding of what China is like. I know where my ancestors’ ancient villages are.”

Some interviewees also expressed doubts about their Chinese identity, especially in the context of the country they are in. P9 said, “But sometimes I feel like I’m not Chinese when I’m in Belgium because my Chinese is not very good. And sometimes I can speak Chinese, but when I come back… it’s like you tell me, you are Chinese.” In addition, since “summer camps” only recruit relevant Chinese youth who meet certain criteria, when asked who is eligible to participate, P6 said, “Anyone can go (to participate in the summer camp), even my Austrian friends can participate, but they cannot speak Chinese, they come over and do not understand anything, it’s meaningless…”

#### 4.1.2 Social conflicts exist in community interactions

Most of the interviewees said that in their daily lives, it is difficult to find like-minded Chinese companions because their parents do not live in Chinese communities. P16 said: “I have an older brother, but he is away from my home. So, I experience the opportunity to interact with others alone. In Italy, many people say that I am always at home and do not have many friends… because I do not live in a city, I usually stay at home and do not go out to play. Coming here, I can meet many people of my age, and I can interact with them in real life. It is good to be able to contact others.” If they have companions for daily interaction, they also have a strong desire to find Chinese-speaking Chinese friends.

Some campers also expressed that in the presence of companions for socializing, they prefer to find Chinese companions. P6 said: “I think I get along better with Chinese friends. There is not much difference with Austrian friends, it’s just that I speak German very well, and sometimes they do not understand what I mean.” P20 said: “I made more friends here than abroad because they are all Chinese, so communication is better.” P21 said: “Because there are not many Chinese people in Portugal, I usually communicate more with foreigners. At that time, I felt that I was forgetting my Chinese a bit, and I felt that I could not forget it because I am Chinese. So, I told my mom that I could speak with Chinese people so that I would not forget it.”

Most of the interviewees expressed that in their daily lives, it is difficult to find like-minded Chinese peers because their parents live in areas that are not Chinese settlements. P16 said: “I have an older brother, but he is outside most of the time, so I am alone. In Italy, many people would ask me why I am always at home and why I do not have many friends… because I live in a non-urban area and usually stay at home. When I came here, I met many peers of the same age and could interact with them in real life, which was great. If they have peers to socialize with, they also strongly desire to find Chinese-speaking friends. Some campers also stated that they prefer to find Chinese peers when they have social interaction with peers. P6 said: “I think I get along better with Chinese friends. There is not much difference with friends in Austria, but my German is very good, and sometimes they do not understand what I mean.” P20 said: “I made more friends here than abroad because they are all Chinese, who are easy to communicate.” P21 said: “Because there are not many Chinese in Portugal, I usually chat more with foreigners. At that time, I felt that I was forgetting my Chinese, and I felt that I could not forget it. I told my mother that I wanted to talk to Chinese people so that I would not forget it.” Many interviewees admitted that social conflicts and negative emotions often arise due to their Chinese identity, as they were born, educated, and living in other countries. P25 mentioned: “I do not know how to communicate with people of my age (in Italy). But when I came to this summer camp, there were many people like me, and I felt very happy. The friends I made here are a bit different from my Italian friends. It’s still in China…” P25 also stated that he does not like negative comments about China from others (referring to Italians), even though they have not lived in China for a long time. “Often Italians see one Chinese person and labelize whole Chinese people. Sometimes, they dislike certain cultural elements, and they make remarks that China is like that. I do not like to talk about ‘what’s good in China’… but (China) feels cleaner. Rome is still very dirty….”

#### 4.1.3 Lack of opportunities to experience traditional culture

“As a stranger in a foreign land, I feel even homesick specifically during traditional Chinese festivals,” as the saying goes. Many campers expressed their desire to return to China for the holidays. P24 said, “I do miss China, especially during the Lunar New Year, but sometimes I just do not have time because we have to study and the holidays are not as long as the winter break in China.” P22 pointed out, “During some festivals, I want to go back home. If I did not go back for the Spring Festival, I would feel lonely without family reunion, and instead just hang out with friends.” P13 said, “For people like us who live outside of China, coming to participate in summer camps is a good opportunity to get to know our own country, to see the scenery here, and to experience the local culture, which I think is great.”

Also, some campers expressed their desire to learn and experience a more diverse Chinese culture, which can only be accessed authentically in China. P6 mentioned, “I like martial arts and dragon dance classes here because they involve sports, and I like sports. Martial arts can only be seen in China.” P25 stated, “Firstly, I came here to learn music, and secondly, to understand Chinese culture. In case some of us need to work and cooperate with others in the future, it is better to have a better understanding of the culture and customs. Not everyone is like me, who grew up in China and then went abroad. They grew up overseas, so they may not have a good understanding of Chinese culture.”

### 4.2 Summer camp enhances Chinese cultural identity

#### 4.2.1 Leisure motivation: from passive to active

Almost all campers showed a significant change in their motivation to participate in activities before and after attending summer camp, from passive to active, and even encouraged their Ethnic Chinese peers to join in. Generally, “parental demands” became the main external motivation for most Ethnic Chinese teenagers to participate in root-seeking summer camps. P29 said, “My dad forced me to come.” P22 said, “They (parents) proposed this idea to me first, and then asked me. I thought it was pretty good, so I signed up.” P12 said, “My parents recommended it to me… I was forced to come by them.” Of course, there are exceptions. P23 said, “At first, he did not support me because he thought that since it lasted for only 10 days, I might not learn anything useful. However, I insisted on joining the camp, he finally let me go.”

However, after being “compelled” to attend the summer camp for the first time, most campers actively sought to participate for a second time, and even more frequently thereafter. P30 stated, “In the beginning, I wasn’t sure what the summer camp was all about, but after participating, I got to make friends from other countries and learn about Chinese culture… I will choose to participate again next year and make more friends.” P28 expressed, “I would participate again, but not in Wenzhou because I would like to go to other places.” P14 claimed, “The first time I came because my parents wanted me to, even though I did not want to. Still, they insisted, and since then I brought along some friends, and we have been going every year willingly.” The data shows that a third of the campers have chosen to participate more than once, among which four members have joined for the fourth time.

Most of the campers became active propagators of recommending the summer camp. Due to the recommendations from peers or personal experience, they either wished to participate again willingly or advocated for their peers to join the summer camp. P25 pointed out, “I can recommend it to him (referring to his Italian Chinese companion).” P14 said, “At first, a friend recommended it. Yeah, they said they were going, and it was a lot of fun.” P3 stated, “This is my second time participating in the summer camp, and the first time was because my classmate asked me to go with him. He has already attended the summer camp many times.”

#### 4.2.2 Leisure experience: from boredom to fun

Summer vacation is boring. Almost all interviewees expressed that they could not “kill” their spare summertime. During this special non-school education time, they were unable to obtain satisfactory socialization with peers, sports activities, and learning development that they could fully immerse themselves in and freely choose from in their home country. The vast majority of campers claimed that they could not find anything or activities to pass the summer vacation in their home country. P14: “I was bored and had nothing to do, so I joined the summer camp to find something to do.” P15 pointed out: “I was bored and had nothing to do… it’s summer vacation, time to relax.”

Summer camp is fun. Most campers expressed that the summer camp activities became an interesting leisure experience to fill the empty box of summer vacation. P14 stated: “If I have to choose what I like, first, it’s because I got to know Chinese traditional culture, like masks and pottery.” P7 stated: “I watched the movie ‘Ip Man’, it was great. There is no one like Ip Man in Austria… I like watching dragon dances, but I do not like performing because the dragon is too heavy, it’s tiring to move it around.” P8 stated: “I like the dragon dance because it looks very beautiful when it’s performed, and the audience enjoys it… I also like to paint with a Chinese brush, and I like to draw bamboo and other things. We went to see those paintings last time, I liked them.”

#### 4.2.3 Leisure interaction: expanding peer circle

If the new generation of Ethnic Chinese are asking themselves “Who am I?” it may be more accurate to say that they are searching for people who are similar to them, rather than questioning their own identity. Feuerbach attributed the origin of self-consciousness to the dyadic interaction between individuals, while Marx and Angels (1965) believed that “man is at first a social being. He is a social being because he recognizes other people as similar to himself. A man called Peter regards himself as a man only because he regards Paul as similar to himself.” In fact, this statement is in agreement with Feuerbach’s view that “in other words, a person can only form self-consciousness through his or her relationship with others. A person discovers himself or herself through others. Self-consciousness cannot be formed in one’s relationship with oneself, but must inevitably be formed in the relationship of opposition and interaction with other-selves (Zhang, 1999).”

Phinney et al. (2001) pointed out that friendship and cultural identity are related, and young people who establish friendships with peers of the same ethnic group have a higher level of ethnic identity. The new generation of Ethnic Chinese finds “peers of the same ethnic group” with similar cultural traits in summer camps. They are searching for friends and see themselves reflected in others. This can be explained psychologically as a need for belonging. In a group, both identification and being identified are equally important. The vast majority of respondents said that finding peers and friends is the most enjoyable thing, and seeking satisfying social activities is also a direct goal of attending summer camp. P8 said: “I have participated four times (in summer camp), and I am quite happy…. My biggest gain is that I have made many friends.” P9 said: “I want to make Chinese friends.” P23: “Being together with classmates, having fun, and laughing is very enjoyable for me. This should be the part I enjoy the most… Then I feel that I have friends in many more places, not just in Greece.”

#### 4.2.4 Context of leisure education

As we all know, education is an important way to enhance cultural identity. In social and practical activities, education and learning are important ways for the formation and development of national cultural identity. The purpose is to make the recognition of the Chinese national culture shift from spontaneous to conscious, from individual to social, and from narrow to comprehensive (Zhan and Wang, 2011) The learning and education of the new generation of Ethnic Chinese are mainly completed through local education, and the Chinese nation has always attached great importance to education. Overseas Chinese also believe that “the important sign of family success is the achievement of their offspring in education, and Ethnic Chinese are therefore known as model minority ethnic groups (Dong, 2016).” Therefore, by creating meaningful educational contexts and through the teaching of summer camp teachers, the influence of Chinese American peers, or their own active experience, they can rethink and arrange the values that guide their behavior and decisions, thereby enhancing their recognition of Chinese culture.

Therefore, it can be further argued that the context of leisure education becomes the stage and soil of cultural identity, and it is the basic color of the educational approach to enhance cultural identity. Brightbill et al. (2009) believes that leisure education can “formally or informally allow people to learn to use their free time to gain self-satisfaction, and make full use of their talents, thereby making free time helpful in improving people’s overall quality of life.” Firstly, through the way of leisure education that is freely chosen, the new generation of Ethnic Chinese can independently choose the skills and knowledge they prefer in their leisure time. Most campers believe that different summer camp themes meet their needs to learn skills, broaden their horizons, and understand the future development of the country, therefore helping them to work in China in the future. P28 pointed out: “If I want to find a job in the future, I can say that I have been to China, learned about Chinese culture, went to many different places, and knew how to communicate with Chinese people and what their personalities are like, which will be very helpful.”

Secondly, summer camps also subtly guide them to experience the long-standing culture and thriving technology of their motherland, and to enjoy the joy of cultural activities in a gentle and serene atmosphere. As mentioned in P9, “I was very happy with yesterday’s singing competition. I do not care if I win or not, I just enjoy it.” P10 says, “I came here to exercise, lose weight, make friends, and participate in activities… I think the most important purpose is not just to play and make friends, but more importantly, through learning, you will know what you can do or what you are interested in, and try it out.”

In addition, there are negative feelings and negative effects of summer camp activities as a result of the mismatch of course difficulty and campers’ cognitive abilities. Difficult courses may undermine campers’ confidence, while simple courses may result in boredom among campers. As mentioned in P15, “I stayed in the classroom, and the teacher told us which area in Wenzhou, but I did not remember any of it because I originally did not come from China and I did not know that place. I did not study Chinese geography or anything like that.” Then they told me a lot at once, and I could not keep up. P20: “There are some courses that, for me, are already very familiar, such as learning Chinese characters and such. I think it’s too boring for me.”

Negative feelings may arise from the words and actions of the camp management and teaching staff. P9 mentioned, “I feel like he always thinks that foreigners from China, like us, are not like Chinese people, and I do not like to hear such comments.” P16: “I might feel that when we have class, our class is very noisy, and many people are sleeping there, and then I feel that the teacher should take care of it and not be too lenient.” P3: “I do not like it when the teacher talks up there and we just listen down below. The teachers in class are good, but they can be a bit boring.”

#### 4.2.5 Creating a culturally immersive experience in a blended setting

The root-seeking summer camp provides a link to original roots through on-site, immersive, and leisurely experiences, offering a platform and opportunity for cultural identity and recognition for the ECNG. The process of recognition, choice, and participation in the summer camp reflects an innate and primal impulse toward Chinese culture, rooted within the consciousness of the ECNG. This impulse creates a “strange” tension between the ECNG and Chinese culture, as they sense a need to seek the cultural origins, attributes, and direction that can address their cultural dilemmas of self-identity and continuity. In the dilemma of self and cultural identity, traditional cultural leisure experiences, such as the root-seeking summer camp, create close ties to personal identity recognition and recognition of Chinese culture for the ECNG.

Data shows that the vast majority of campers understand the significance of the root-seeking journey. The purpose of the journey is to “understand Chinese culture and history, and to find one’s roots.” At the same time, some people also understand that a root-seeking journey generally means going out to play, learn, and travel, and through these fun and relaxing activities, they can find their “origins.” P9 expresses, “When we leave China, sometimes we lose our sense of cultural identity. Now that I have returned, I feel it again.”

The vast majority of people knew about the root-seeking journey before participating in the project and understood the meaning of the root-seeking journey.” P13 said, “I did find out about the root-seeking journey, but I did not pay much attention to it at first. I mainly heard that I could learn more about China and it sounded interesting…. If I were to care about it, maybe it’s… but I did not think about what the name meant at first. Later, someone explained that a root-seeking journey means that some people may be from Wenzhou, so it’s about finding their origins or something.”

The positive feelings and experiences that ECNG have during cultural activities such as outdoor activities, singing competitions, tea art, and Chinese traditional dress experiences, can stimulate their active cultural feelings and experiences. P15 pointed out, “The happiest thing is to go out and play… the classes that you experience personally.” P16 also mentioned, “I think it’s better to go outside… because in the classroom everything is a bit rigid…there was a traditional Chinese experience before, experiencing the traditional things of China, like tea art and Hanfu, I do not know if you know about it.”

## 5 Discussion

This study focuses on ECNG and examines the influence of participating in root-seeking Summer Camp” leisure activities on their identification with Chinese culture. The results show that the ethnic identity of ECNG is not clear, with conflicts in their community relationships. Opportunities for experiencing traditional culture are scarce, which reflects the twists and turns and conflicts in their identification with Chinese culture. The leisure activities of the summer camp mobilized the leisure motivations, experiences, and interactions of the ECNG, constructed a mechanism for enhancing cultural identity, and provided a stage and opportunity for developing identification with Chinese culture through creating a context of leisure education and cultural experience.

Firstly, the study found that the ethnic identity of ECNG is not clear with conflicts in their community relationships, and opportunities for experiencing traditional culture are scarce. Especially during their adolescence, they have a stronger need for exploring their ethnic identity, and group affiliation and experiencing the traditional culture of their ancestral country. This echoes the research conclusions of scholars such as Xia (2020) and Zheng (2012). To summarize, compared with their parents, they live in the host country’s cultural environment, receive education from the host country’s schools, and are equipped with characteristics reflecting the host country’s spirit, culture, values, and concepts. At the same time, their physical characteristics such as appearance and skin color, differ from those of the host country, and they are home-schooled by Chinese parents and are influenced by the overseas Chinese community. Although they have long-term overseas living experiences, they still feel confused about their identification with ethnicity, community, and culture.

Secondly, in addition to supporting previous research findings, this study found that the root-seeking summer camp helps improve the cultural identity of the new generation of Ethnic Chinese (Yang and Zheng, 2009; Lin, 2015). The difference between this study and previous studies is that previous viewpoints suggested that “enhancing the cultural identity of the new generation of Ethnic Chinese is mainly achieved through Chinese language education (Wang and Lan, 2013)” and “family education and Chinese language education play a crucial role in their ancestral cultural identity (Zhang, 2017).” This study, on the other hand, found that through theoretical lenses of leisure and identity, the summer camp’s leisure activities construct the internal mechanism of cultural identity for the new generation of Ethnic Chinese through three dimensions, namely, leisure motivation (McAdams, 1992), leisure experience (Martin, 1995), and leisure interaction (Barth, 1998). Constructivism posits that multiple factors such as motivation, experiential practice, and ethnic interaction jointly influence cultural identity. This study found that ECNG exhibited a clear change in their willingness to participate in leisure activities after attending the summer camp, with internal motivation significantly higher than external motivation; they also experienced more positive leisure experiences and had more opportunities for leisure interactions with Chinese members, thus enhancing their cultural identity with their ancestral country.

Furthermore, this study found that the summer camp activities create an educational field for ECNG to freely explore their potential, as well as a cultural field for free experience with full engagement. They can choose to learn various skills and vividly experience and understand their ancestral culture. The theory of field proposed by Bourdieu suggests that fields include cultural fields, educational fields, etc., which cover language symbols, power, and personal strategies (Swartz, 2006). The cultural identity game of ECNG occurs between the local culture and the ancestral culture in fields such as power, education, and media, which are the most intense. The context created by the root-seeking summer camp has both an educational and cultural influence, and the common feature of these two fields is a leisure context. In the context of leisure and the ever-changing social process, leisure actors constantly redefine themselves based on social identity, maintain a satisfactory identity, negotiate and revise unsatisfactory identities, and establish identification in the process of social assimilation through positioning themselves and others (Peng, 2018).

Thirdly, this study suggests that the main organizers of the root-seeking summer camp adjust their management and teaching behaviors, and establish a concept of cultural inclusivity. For one thing, it is particularly important to set appropriate levels of difficulty for cultural experience projects. Psychologist Csikszentmihalyi proposed the theory of “flow,” which refers to an optimal experience that can be achieved during “work” or “leisure” activities. The attainment of a smooth experience is closely related to the “challenge level,” if the difficulty level far exceeds the individual’s ability, it will create excessive anxiety, while if the difficulty level falls below the individual’s skill level, it may trigger boredom.

For another thing, the organizers and direct managers of the summer camp should create a relaxed and free atmosphere, especially in affairs related to personal values and identity, and pay more attention to communication skills. Kelly (1987) proposed that leisure and play may be important factors in this process (personal identification). In a relaxed, free, and tolerant atmosphere of leisure activities, people can do whatever they want to do and freely explore, and continue to develop themselves.

## 6 Conclusion

The purpose of this study was to explore how root-seeking Summer Camp helps to enhance the cultural identity of the ECNG. The study revealed that the summer camp constructed a mechanism to enhance the cultural identity of the ECNG through three dimensions, namely, leisure motivation, leisure experience, and leisure interaction, by creating a context for leisure education and cultural experience and providing a platform for the development of cultural identity.

This study is the first systematic qualitative research that focuses on the role of the root-seeking Summer Camp in enhancing cultural identity among the ECNG with a complex cultural background. It also echoes the research of Kelly et al., who view leisure as a private place for individual self-expression and identity development (Kivel and Kleiber, 2000).

The theoretical contribution of this study is to construct the intrinsic mechanism of leisure motivation, leisure experience, and leisure interaction through the root-seeking Summer Camp as a carrier of leisure activities, to create a context for education and cultural experience, to enhance cultural identity, and to contribute to the theoretical research of leisure and identity. The empirical value of this study is that, firstly, the organizers and managers of the summer camp, should continue to strengthen the organization of summer camp activities and attract more ECNG to participate. Secondly, organizers should carefully design courses with different levels of difficulty and design more outdoor experiential activities according to the preferences of ECNG.

## 7 Limitations and future research

Despite the illuminating discoveries presented in this study, it also encapsulates several limitations. Primarily, the sample size employed is comparatively modest. Additionally, divergences might exist in the interpretation of participating in root-seeking summer camps to enhance cultural identity across different national cultural backgrounds. The mechanisms of cultural identity revealed in this research may not necessarily hold explanatory power for other nations. Consequently, future researchers are encouraged to engage in investigations encompassing a broader array of samples to derive more substantiated conclusions. Furthermore, should a longitudinal study on these campers’ identification with Chinese culture be executed, the discourse could potentially be enriched considerably. Last but by no means least, while academic discourse may predominantly focus on how the new generation of immigrants integrate into local cultures, the preservation of their original cultural identity merits equal attention.

## Data Availability

The raw data supporting the conclusions of this article will be made available by the authors, without undue reservation.
